# Genetic and epigenetic alterations in differentiated thyroid carcinoma

**Published:** 2013-12-25

**Authors:** AC Brehar, FM Brehar, AC Bulgar, C Dumitrache

**Affiliations:** * “C.I. Parhon” National Institute of Endocrinology, Bucharest; ** “Bagdasar Arseni” Emergency Hospital, Bucharest; *** ”Carol Davila” University of Medicine and Pharmacy, Bucharest

**Keywords:** differentiated thyroid carcinoma, genetic and epigenetic alterations

## Abstract

Abstract

Differentiated thyroid carcinoma (DTC) has a favorable prognosis, but it is important to identify those patients who have a high risk of progressive disease and DTC-related death at the time of diagnosis. Analyzing genetic and epigenetic alterations in thyroid cancer may play a role in tumor diagnosis, prognostic and therapeutic strategies.

## Introduction

Thyroid cancer (TC) is the most common malignancy of endocrine organs and its incidence has been steadily increased [**1**]. There are two main types of thyroid cancer: follicular cell-derived type and medullary type. The follicular cell-derived cancer develops from thyroid follicular epithelial cells and has several subtypes: well-differentiated papillary carcinoma (PTC) and follicular carcinoma (FTC), poorly differentiated carcinoma, and anaplastic (undifferentiated) carcinoma. The medullary thyroid cancer derived from parafollicular C cells (MTC) [**2**]. Follicular adenoma (FA) is a benign tumor that may be a precursor for some follicular carcinomas. Less-differentiated thyroid cancers, poorly differentiated carcinoma and anaplastic carcinoma (ATC), can develop de novo, or through a process of stepwise dedifferentiation of papillary and follicular carcinomas [**2**].

PTC represents 80% of all thyroid malignancies, FTC approximately 15% of cases, MTC represents 3% of thyroid malignancies, and ATC, the most aggressive form of TC, represents 2%. PTC is more frequent in childhood and in adults <50 years, FTC in patients <60 years, and the ATC in patients between 60–70 years [**3**].

Variants of PTC are classical form with papillary architecture, follicular variant, oncocytic variant (or Hurthle-cell variant), tall-cell variant or solid and cribriform types, each with distinct patterns of growth and clinical behaviors [**3**]. Variants of FTC include oncocytic (Hurthle-cell) and clear-cell types.

There are some risk factors that contribute to the development of thyroid carcinoma: radiation exposure, reduce iodine intake, thyroiditis, hormonal factors and family history.

Differentiated thyroid cancer (DTC), which includes PTC and FTC, is generally curable. However, recurrences occurs in up to 40% of patients and are difficult to manage because of losing radioiodine (RAI) avidity and becoming unresponsive to (131I) treatment [**4**]. Therefore, it is important to understand the genetic and epigenetic alterations in DTC in order to develop molecular based diagnostic and therapeutic strategies [**5**].

Molecular biology of thyroid cancer

**Fig. 1:**
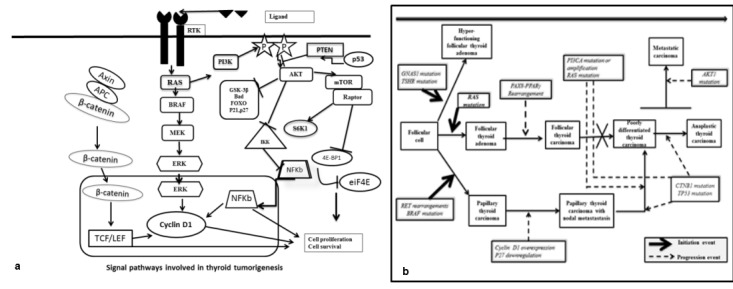
Fig. 1a Signal pathways in thyroid tumorigenesis. 1b. The stepwise of thyroid carcinogenesis - after Giuseppe Viglietto and Carmela De Marco (2011). Molecular Biology of Thyroid Cancer, Contemporary Aspects of Endocrinology [**8**]

Thyroid cancer initiation and progression occurs through gradual accumulation of various genetic and epigenetic alterations that involve the activation of MAPK and PI3K-AKT signaling pathways [**6**]. MAPK activation is important for tumor initiation and the PI3K/AKT signaling pathway is necessary for the progression and dedifferentiation of thyroid cancer. The mutated genes encode cell-membrane receptor tyrosine kinase RET and NTRK1 and intracellular signal transducers BRAF and RAS [**6**]. PTEN is a phosphatase that acts as a suppressor of PI3K/AKT pathway. Beta-catenin is a part of a cytoplasmic complex also containing APC (adenomatosis polyposis coli) and axin, which is regulated by glycogen synthase kinase-3(GSK3b). When the complex is phosphorylated by GSK3b it undergoes ubiquitination and degradation. AKT phosphorylates and inactivates GSK3b releasing b-catenin from the complex, allowing it to be translocated to nucleus where b-catenin activates T-cell-specific transcription factor/lymphoid enhancer binding factor (TCF/LEF) target genes such as cyclin D1 and myc, which promote cell proliferation [**7**] (**Fig. 1**).

 The mechanisms involved in the pathogenesis of thyroid cancer are genetic and epigenetic.

 Genetic events

 I. Mutations: Nuclear gene mutations: and mitochondrial gene mutations

 II. Gene rearrangements

 III. Loss of heterogeneity (LOH)

 Epigenetic events: DNA methylation, histone modification and gene silencing through microRNA (miRNA). 

 I.Mutations 

 1.Nuclear gene mutations 

 1)BRAF mutations The MAPK pathway is a intracellular signaling pathway that plays a role in cell proliferation, differentiation, survival, and in tumorigenesis (when it is aberrantly activated) [**5**]. The activators of this pathway in thyroid cancer include RET/PTC rearrangements, Ras. mutations and BRAF mutations [**5**].

 There are three Raf protein kinases: A-Raf, B-Raf (BRAF the most potent activator of the MAP kinase pathway), and C-Raf. The T1799A point BRAF mutation (a V600E amino acid change in the BRAF protein resulting in constitutive and oncogenic activation of the BRAF kinase) represents more than 90% of all BRAF mutations in human cancer and is the most common genetic alteration in thyroid cancer (a somatic rather than germline mutation in thyroid cancer) [**9**]. Other types of BRAF mutations are rarely described in thyroid cancer: the BRAF V599ins, BRAF V600E+K601del, BRAF K601E, AKAP9-BRAF, and V600D+FGLAT601- 605ins, which result from an insertion of 18 nucleotides at nucleotide T1799 [**7**].

 The V600E BRAF mutation uniquely occurs in 45% of PTC, 25% of ATC and does not occur in FTC or other types of thyroid tumors and is highly prevalent in adult PTC, but is infrequent in childhood thyroid cancer as well as in radiation-induced thyroid tumors and is more commonly seen in recurrent PTC (prevalence of 80%–85%) versus primary PTC (prevalence of 45%) [**10**-**12**]. Activating BRAF mutations in papillary thyroid carcinomas have been linked to aberrant methylation of several tumor-suppressor genes, including TIMP3, SLC5A8, DAPK and RAR 2 that corresponds with the dedifferentiation of PTC which is correlated with signs of aggressive behavior: extrathyroidal invasion, lymph node metastasis and advanced tumor stage at diagnosis. The aggressiveness conferred by the BRAF mutation in thyroid tumorigenesis occurs even in low stage and papillary thyroid microcarcinomas because BRAF V600E is associated with vascular endothelial growth factor (VEGF) over-expression [**13**,**14**].

 2)RAS mutation RAS is a kinase that is upstream of BRAF and mutant RAS constitutively activates the MAPK and PI3K/AKT signaling pathways. Three RAS (H−, N−, and K−) forms exist. RAS mutation occurs in 10–20% of PTC, 40–50% of FTC, and in more than 50% ATC. These mutations always localize to either codon 12, 13, or 61 [**15**]. Ras and BRAF mutations were mutually exclusive in differentiated PTC, suggesting that, like BRAF mutation, Ras mutation is also able to independently cause PTC through the MAP kinase pathway. Ras mutants also activate the PI3K/Akt pathway in human cancer Through the p110 catalytic subunits of PI3K class I that contains a Ras-binding site [**5**]. Different isoforms of Ras mutants may play a different role in activating the PI3K/Akt and MAP kinase pathway pathways in human cancer [**16**]. The N-Ras mutant is a preferential activator of the PI3K/Akt pathway, whereas K-Ras is a preferential activator of the MAP kinase pathway [**17**]. RAS-positive follicular adenomas may serve as a precursor for RAS-positive follicular carcinomas and the follicular variant of papillary carcinomas. Furthermore, RAS mutations may predispose well-differentiated cancers to dedifferentiation and anaplastic transformation [**18**].

 3)PTEN gene PTEN is a tumour suppressor gene localized to chromosome 10q23 that has protein and lipid phosphatase activity and inhibits PI3K/AKT pathway. Deletion of the PTEN locus at 10q23 (20–60% of thyroid malignancies), and silencing of PTEN by aberrant promoter methylation (>50% of FTC) enhance PI3K signaling and is associated with the progression of thyroid tumors [**19**]. Mutations of PTEN gene have been reported in about 7% of FTCs and 15% of ATCs cases, whereas they have not been found in follicular adenomas [**20**].

 4)TP53 Gene TP53 gene (gene located on chromosome 17) encodes a tumor suppressor and mutation of TP53 could be responsible for the loss of differentiation observed during tumor progression. In thyroid cancer these mutation is present in approximately 60%–80% of ATCs, in 30% of poorly diﬀerentiated tumors, and only rarely in FTCs and PTCs mostly involving the exons 5–8 of the gene [**21**].

 5)CTNNB1 (β−Catenin) Gene CTNNB 1 gene (gene on chromosome 3ρ22–21.3), encodes β−catenin, a cytoplasmic protein that plays a role in cell adhesion and transcription being an intermediary in the wingless signaling pathway (WNT). Point mutations at exon 3 of CTNNB1 gene have been found in 25% of poorly diﬀerentiated carcinomas and 66% of ATCs, respectively, but not in well-diﬀerentiated carcinoma [**22**].

 2. Mitochondrial gene mutations:

 Gene NDUFA13 (also known as GRIM 19) encodes a protein that regulates cell death and promotes apoptosis, and also affects mitochondrial metabolism by serving as an essential component of complex I of the mitochondrial respiratory chain. Mutations of the gene NDUFA13 (also known as GRIM 19) have been identified in oncocytic thyroid tumors.In one study, somatic missense mutations in NDUFA13 were found in 10–20% of oncocytic follicular carcinoma and the oncocytic variant of papillary carcinoma. These mutations may disrupt the function of this antiapoptotic tumor suppressor gene and promote tumorigenesis [**6**].

 II.Gene Rearrangement Gene rearrangements lead to a novel protein with oncogenic properties. There are some trasnlocations describes in thyroid carcinomas:

 1.PAX8/PPARΥ Rearrangement represents a chromosomal translocation t(2;3)(q13;ρ25) which fuses PAX8, a thyroid specific transcriptor factor to PPARΥ a nuclear hormone receptor involved in the differentiation of cells especially adipocytes. This rearrangement represents an early event in the development of FA and FTC. It was described in FTC (36%), FA (11%) and follicular variant of PTC (13%) [**23**-**25**].

 2.RET/PTCs In thyroid follicular cells, RET is not expressed or it is expressed at very low levels, and RET activation can occur through chromosomal rearrangements that result in the in-frame fusion of part of RET intracellular domain coding region (including that coding for the TK from residue E713 and the carboxy-terminal tail) with the 5 end of heterologous genes [**26**]. The RET proto-oncogene is located on chromosome 10q11.2 and encodes for a transmembrane tyrosine-kinase receptor involved in the control of cell differentiation and proliferation. Four different ligands have been reported up to now: glial derived neurotrophic (GDN) factors, Neurturin (NRTN), Artimin (ARTN), and Persepin (PSPN). All these ligands induce RET activation through the binding to speciﬁc coreceptors [**27**]. 13 different types of RET/PTC rearrangements have been reported and all of them are the result of the fusion of the RET tyrosine-kinase (TK) domain with different genes, with constitutive dimerization of the RET TK domain which determines an uncontrolled proliferation of the follicular cells harboring the RET /PTC rearrangement and the development of malignancy.

 The reported RET /PTC prevalence in thyroid tumors varies greatly in different series and this difference can be attributed to ethnical and geographic variations as well as to the different sensitivities of detection methods and tumor heterogeneity [**27**].

 Clonal RET /PTC rearrangements occur in about 20% of PTC and are speciﬁc for this tumor Non-clonal RET /PTC rearrangements have been found not only in PTC but also in 10–45% of thyroid adenomas and other non-neoplastic thyroid lesions and Hashimoto’s thyroiditis [**27**]. The most common gene rearrangement products are RET/PTC1 (inv(10)(q11.2;q21)) and RET/PTC3 (inv(10)(q11.2;q10)). Both involve inversion of the long arm of chromosome 10, generating a fusion between RET and either histone H4 (histone protein in nucleosome) or nuclear receptor coactivator 4 (NCOA4) gene, respectively, for RET/PTC1 and RET/PTC3 and are paracentric, intrachromosomal inversions and RET/PTC2 and nine more recently discovered types of RET/PTC rearrangements are all interchromosomal rearrangements [**26**, **27**].

 RET/PTC oncoproteins are believed to take part in several mechanisms that allow tumor growth and spread, including angiogenesis, invasion, metastasis, and immune escape. RET/PTC induces these signiﬁcant phenotypic changes oriented toward neoplastic transformation affecting the tumor microenvironment [**26**]. It was proposed that RAS/BRAF and/or PI3K/AKT pathways are required for cellular transformation and that the additional proinﬂammatory pathways of RET/PTCs shape the features of the growing tumor [**26**].

 3.AKAP9/BRAF (inv(7)(q21–22q34)) rearrangement is an inversion of chromosome 7q generating fusion between BRAF and AKAP9 (A-kinase anchor protein 9 gene) containing BRAF kinase domain without the N-terminal auto-inhibitory domain. This fusion protein has elevated kinase activity. BRAF mutation is common in sporadic PTC, while this novel AKAP9/BRAF (inv(7)(q21–22q34)) rearrangement occurs in radiation-induced thyroid carcinomas [**28**].

 4.Rearrangements of NTRK1 gene (TRK rearrangements) The NTRK1 gene on chromosome 1q22 can be fused to at least three different partner genes located on the same or different chromosomes. The other less common (<12%) gene rearrangement in PTC involves the fusion between the 3' terminal sequences encoding the kinase domain of NTRK1 (neurotrophic tyrosine kinase receptor type 1) on chromosome 1 and 5' terminal sequences of various genes resulting in activated TRK oncogenes. The most frequent fusion product is between NTRK1 and TMP3 (non-muscle tropomyosin) [**7**].

 III. LOH represents the loss of the normal function of one allele of a gene in which the other allele was already inactivated at the somatic or germline level. LOH is detected on average in 6%–12% of follicular adenomas and in 30%–50% of FTCs. The chromosomal regions most frequently involved are located on chromosomes 2p, 3p, 9q, 9p, 10q, 11p, 17p, and 15q. The frequency of LOH has been correlated with the aggressiveness of the tumor and the presence of relapse in patients with FTC [**29**]. LOH is present more often in thyroid oncocytic tumors.

 Epigenetic mechanisms. The mechanisms of epigenetic regulation are the following: DNA methylation, chromatin remodeling, through repositioning or reconﬁguration of the nucleosomes, histone modification and gene silencing through microRNA (miRNA).

 DNA methylation involves covalent attachment of a methyl group at position 5 in the cytosine ring by DNA methyltransferases that results in a methyl cytosine. and plays central role in various cellular and biological processes like gene expression, generation of defense mechanism against various viral sequence as well as transposable elements silencing [**30**,**31**].

Histone modification includes methylation, acetylation, phosphorylation, ubiquitination and contribute to the onset and progression of tumorigenesis.

 miRNA acts as either tumor suppressor gene or oncogene and targets of miRNA are genes which are involved in various cellular and differentiation process [**32**].

 DNA metylathion Various genes involved in the control of cell proliferation and invasions as well as genes speciﬁc for thyroid differentiation are epigenetically silenced in thyroid cancer. Cumulative epigenetic alterations play a role in the sequential progression from indolent WDTC to metastasizing carcinomas, through the spectrum of poorly differentiated to undifferentiated thyroid carcinoma [**32**].

 Methylated genes involved in the control of proliferation and invasion are the following: P16INK4A (Cyclin dependent kinase inhibitor) with a prevalence of 30% of thyroid cancer [**33**]; RASSF1A (Microtubule stabilization) describe in 30% of thyroid cancer [**34**,**35**]; PTEN (Modulator of the PI3K/AKT pathway) observed in 50% of PTC, 100% of FTC [**36**], Rap1GAP (Rap1 GTPase-activating protein) in 72% of PTC, 38% of FTC [**37**]; TIMP3 (Tissue inhibitor of metalloproteinase) in 53% PTC (associated with BRAF mutation) [**38**]; DAPK (Ca/calmodulin-dependent ser/thr kinase protein), in 34%PTC (associated with BRAF mutation) [**39**]; RARβ2 (Negative regulator of cell growth) in 22%PTC (associated with BRAF mutation) [**39**]; E-cadherin/CDH1 (Regulator of cellular adhesion) in many CV-PTC and 3 out of 5 DSV-PTC [**40**,**41**]; CITED1 (Cbp/p300 Interacting Transactivators with glutamic acid [E] and aspartic acid [D]-rich C-terminal domain) in PTC [**42**]; hMLH1 (DNA repair gene) 38% in PTC (associated with BRAF mutation) [**43**].

 Methylated thyroid specific genes are the following: Na/I symport, (Sodium iodide symporter (NIS)) in 22% PTC [**44**,**45**]; TSH receptor (Thyroid-stimulating hormone receptor) in 34–59% of thyroid cancer [**46**,**47**]; Pendrin (SLC5A8/ Apical Iodide Transporter (AIT)) in 33% PTC (associated with BRAF mutation) [**39**]; TTF-1 (Thyroid transcription factor-1) in 60% of the undifferentiated carcinomas [**48**].

 A close association between BRAF mutation and aberrant methylation of several tumor-suppressor genes in PTC, including the genes for tissue inhibitor of matrix metalloproteinase-3 (TIMP3), death-associated protein kinase (DAPK), and retinoic acid receptor β2 (RARβ2) has been reported [**38**]. This association correlated with high-risk clinicopathological characteristics of PTC, including extra-thyroid invasion, lymph node metastasis, and advanced disease stages [**36**]. Methylation of the RASSF1A gene was inversely associated with the BRAF mutation in PTC.

 Histone modifications CpG hypermethylation in the promoter region of the thyroid transcription factor-1 (TTF-1), which is essential for thyroid organogenesis, concurrently with increased dimethyl-H3-K9 and decreased acetyl-H3-K9, has been observed in a subset of thyroid carcinoma cells that had lost TTF-1 expression [**48**]; moreover, it has recently been demonstrated that the enhancer of zeste homolog 2 (EZH2), a histone lysine methyltransferase belonging to the polycomb group protein family, is speciﬁcally overexpressed in ATC, and it directly contributes to transcriptional silencing of PAX8 gene and ATC differentiation [**50**].

 miRNAs MicroRNAs (miRNAs) are endogenous single-stranded noncoding RNAs of about 22 nucleotides which suppress gene expression by selectively binding to the complementary 3 untranslated region (3'-UTR) of messenger RNAs (mRNAs) through base-pairing. They play important roles in multiple biological and metabolic processes, such as cell differentiation, proliferation and survival [**50**]. The overexpression of specific miRNAs could lead to the repression of tumor suppressor gene expression, and conversely the downregulation of specific miRNAs could result in an increase of oncogene expression. In thyroid tumors 32% of all known human miRNAs are up-regulated and 38% down-regulated with more than a 2-fold change as compared to normal tissues [**51**]. The miRNA expression profile presents a significant variability between different kinds of thyroid cancers, even if they originate from the same type of thyroid cells, between tumors at different stages of malignancy, and between primary tumors and metastases [**50**-**52**].

 MiRNAs involved in PTC are the following: miR−146,−221,−222,−21,−181a,−155,−213,−181b,−31,−172,−34a,−223,−224,−187,−146b,−220 (down-regulated) and miR−26a−1,−345,−138,−319,−218,−300,−292,−30c (up-regulated). MiRNAs involved in FTC are the following: miR−197,−346,−187,−221,−222,−224,−203,−183,−339,−31 (down-regulated) [**53**].

## Conclusions

Genetics and epigenetic alterations in thyroid cancer are interlinked and represent the signature of the disease that could be used as biomarkers in early detection and are potential targets of therapeutic genetic strategies.
